# Supplementary material for: Genome-Wide Identification of the CAT Genes and Molecular Characterization of Their Transcriptional Responses to Various Nutrient Stresses in Allotetraploid Rapeseed
Source: Int J Mol Sci. 2024 Nov 25;25(23):12658. doi: 10.3390/ijms252312658 (PMC11640766; doi:10.3390/ijms252312658)
Supplement: Supplementary file 1 [file ijms-25-12658-s001.zip › Supplementary pictures.pdf]

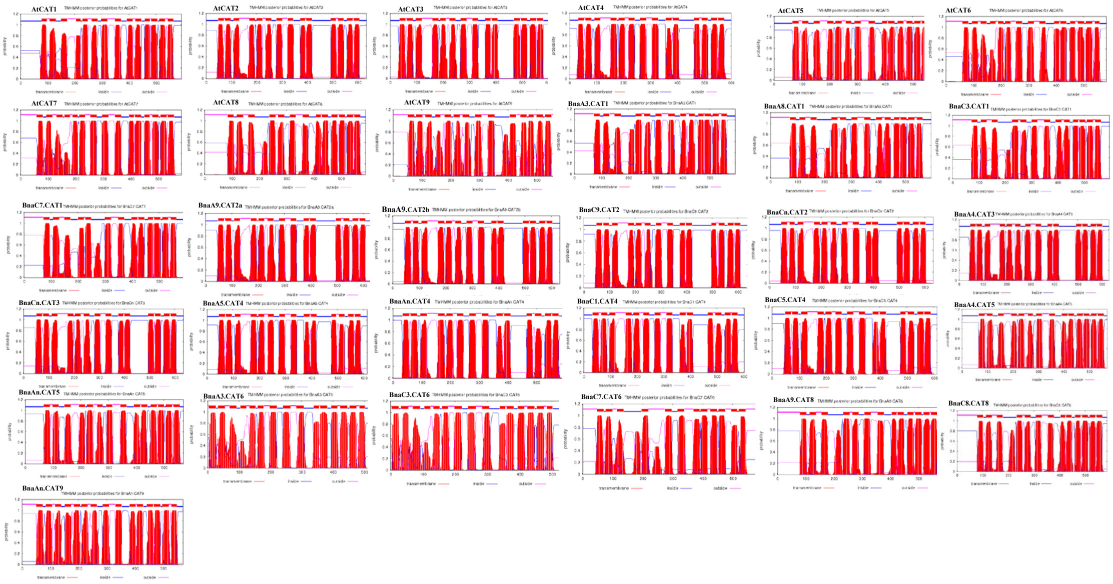

Figure S1 Trans-membrane characterization of the cationic amino acid CAT proteins in *Arabidopsis thaliana* and *Brassica napus.* The TMHMM (http://www.cbs.dtu.dk/services/TMHMM/) tool was used to predict the transmembrane topology of the AtCAT and BnaCAT proteins.

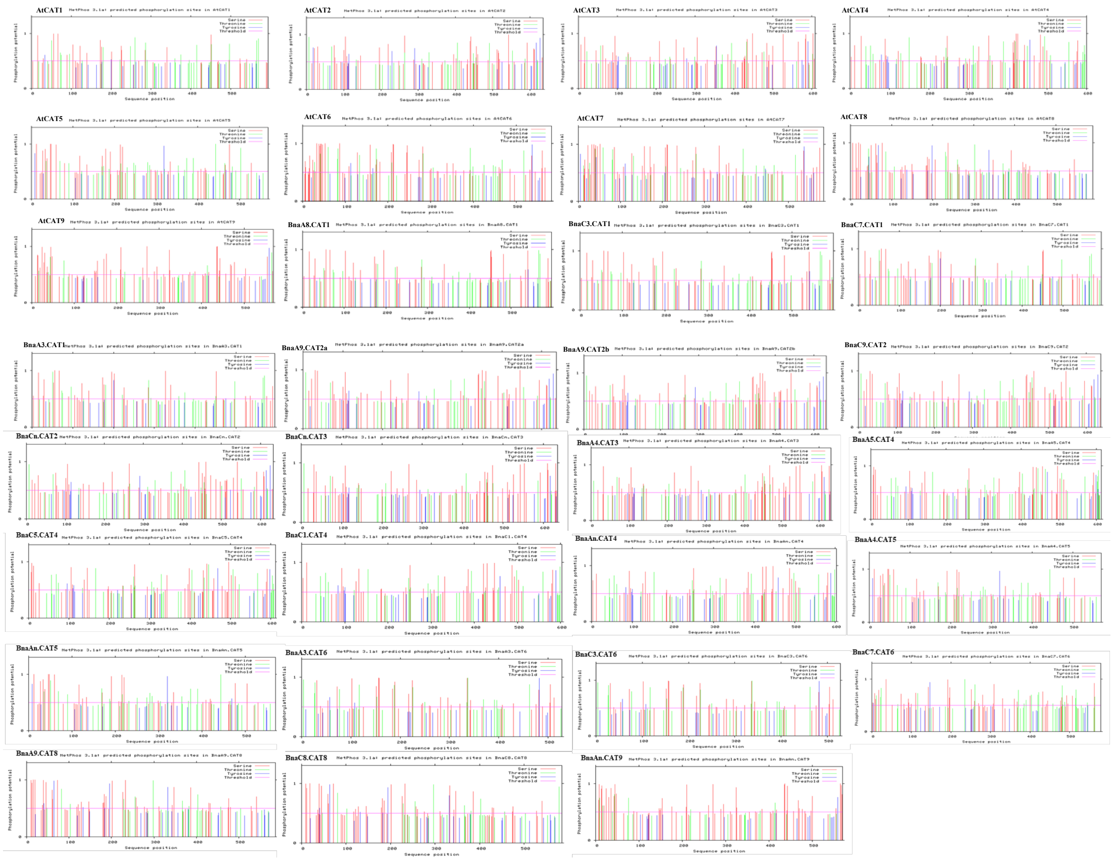

Figure S2 Phosphorylation sites of the cationic amino acid transporter (CAT) proteins in *Arabidopsis thaliana* and *Brassica napus*. The NetPhos (http://www.cbs.dtu.dk/services/NetPhos/) 3.1 server was used to predict the presence and location of phosphorylation sites in amino acid sequences of the AtCAT and BnaCAT proteins.

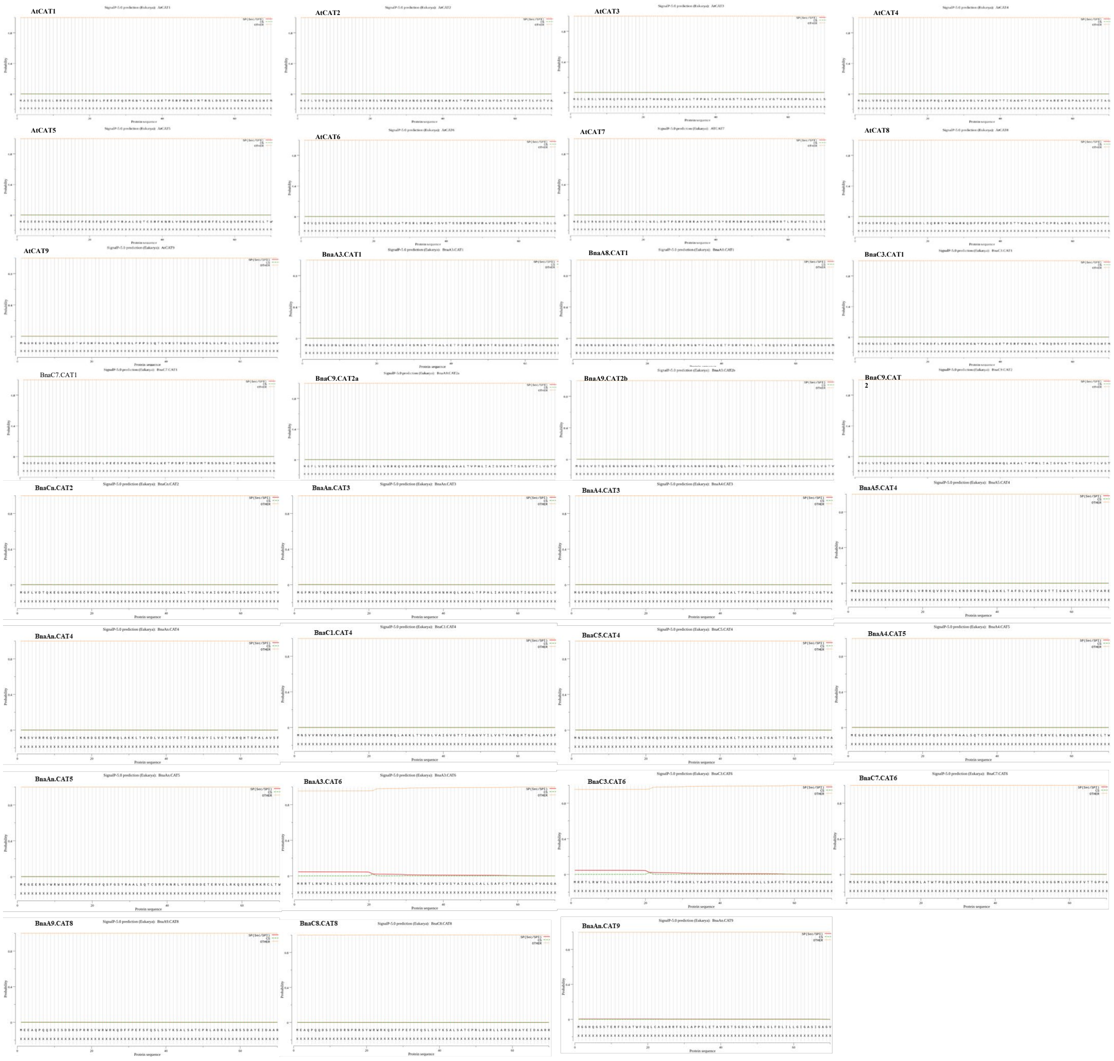

Figure S3 Characterization of signal peptides of the cationic amino acid (CAT) proteins in *Arabidopsis thaliana* and *Brassica napus.* The SignalP (http://www.cbs.dtu.dk/services/SignalP/) 4.0 server was used to predict the presence and location of signal peptide cleavage sites in amino acid sequences of the AtCAT and BnaCAT proteins.

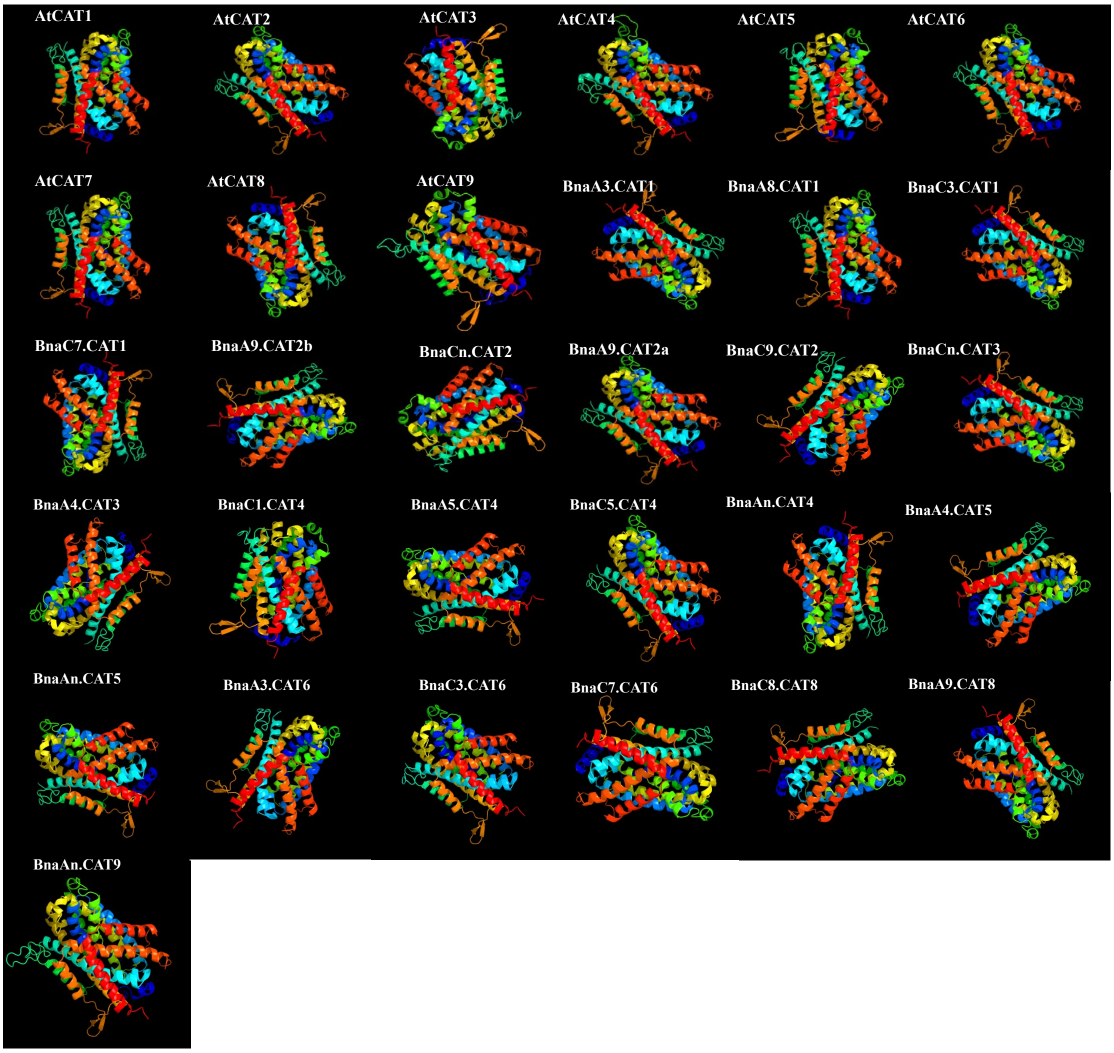

Figure S4 The secondary structure of the cationic amino acid proteins in *Arabidopsis thaliana* and *Brassica napus*. The Phyre2 (http://www.sbg.bio.ic.ac.uk/phyre2/webscripts/jobmonitor) was used to predict the secondary structure in amino acid sequences of the AtCAT and BnaCAT proteins.

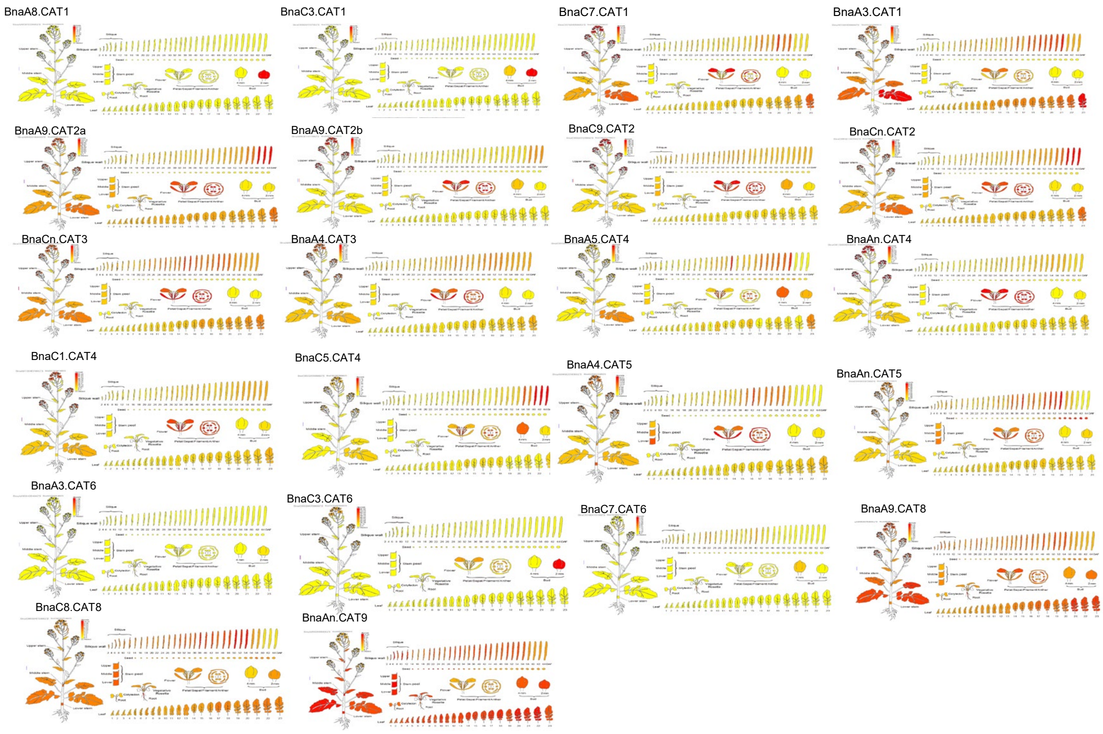

Figure S5 Tissue-specific expression patterns of the *cationic amino acid transporter* (*CATs*) in *Brassica napus*. The red and yellow color indicates relative high and low expression levels of *BnaCATs*.
