# Supplementary material for: Genome-Wide Identification of the CAT Genes and Molecular Characterization of Their Transcriptional Responses to Various Nutrient Stresses in Allotetraploid Rapeseed
Source: Int J Mol Sci. 2024 Nov 25;25(23):12658. doi: 10.3390/ijms252312658 (PMC11640766; doi:10.3390/ijms252312658)
Supplement: Supplementary file 1 [file ijms-25-12658-s001.zip › Supplementary tables.pdf]

Table S1. Molecular characterization of the cationic amino acid transporters (*CATs*) in *Arabidopsis thaliana* and *Brassica napus*

Gene ID	Gene Name	pI	MW (kDa)	II	GRAVY	TM Domains	Subcellular Localization
AT4G21120.1	*AtCAT1*	7.94	64.84	31.53	0.519	14	PM
BnaA08g09810D	*BnaA8.CAT1*	6.83	64.86	29.36	0.537	13	PM
BnaC07g36580D	*BnaC7.CAT1*	7.6	64.81	27.77	0.512	13	PM
BnaC03g64380D	*BnaC3.CAT1*	6.6	64.83	28.97	0.528	13	PM
BnaA03g58530D	*BnaA3.CAT1*	7.12	64.76	28.21	0.519	13	PM
AT1G58030.1	*AtCAT2*	5.58	67.11	32.07	0.689	14	Vac
BnaA09g12110D	*BnaA9.CAT2a*	5.79	67.58	31.64	0.614	14	PM
BnaA09g53040D	*BnaA9.CAT2b*	6	67.13	30.14	0.643	14	PM
BnaCnng25140D	*BnaCn.CAT2*	5.91	67.05	29.6	0.642	14	PM
BnaC09g12080D	*BnaC9.CAT2*	5.76	67.80	32.79	0.604	14	PM
AT5G36940.1	*AtCAT3*	8.07	65.28	33.86	0.682	14	ER
BnaCnng50730D	*BnaCn.CAT3*	6.56	68.03	35.11	0.614	14	PM
BnaA04g07800D	*BnaA4.CAT3*	6.18	67.59	34.65	0.637	14	PM
AT3G03720.2	*AtCAT4*	5.79	63.63	33.64	0.723	14	Vac
BnaC01g40540D	*BnaC1.CAT4*	5.67	64.14	34.36	0.654	14	PM
BnaA05g32770D	*BnaA5.CAT4*	6.77	65.48	30.84	0.653	14	PM
BnaC05g48070D	*BnaC5.CAT4*	7.14	65.36	30.26	0.64	14	PM
BnaAnng23630D	*BnaAn.CAT4*	5.56	64.25	34.56	0.643	14	PM
AT2G34960.1	*AtCAT5*	9.03	62.63	31.42	0.543	14	PM
BnaAnng19530D	*BnaAn.CAT5*	8.5	62.73	31.03	0.546	14	PM
BnaA04g20450D	*BnaA4.CAT5*	8.5	62.71	31.18	0.546	14	PM
AT5G04770.1	*AtCAT6*	8.85	62.80	37.18	0.662	15	PM
BnaA03g01440D	*BnaA3.CAT6*	8.83	56.98	37.02	0.791	15	PM

BnaC03g01740D	*BnaC3.CAT6*	8.84	56.92	36.16	0.795	15	PM
BnaC07g34120D	*BnaC7.CAT6*	9.02	63.46	29.96	0.62	12	PM
AT1G17120.1	*AtCAT8*	8.64	64.86	33.6	0.594	13	PM
BnaC08g37970D	*BnaC8.CAT8*	8.52	64.02	33.83	0.585	13	PM
BnaA09g45150D	*BnaA9.CAT8*	8.33	64.18	36.89	0.594	13	PM
AT1G05940.1	*AtCAT9*	7.55	60.17	41.28	0.753	14	Vac/ER
BnaAnng20490D	*BnaAn.CAT9*	6.59	60.43	41.71	0.772	15	PM/ER

Table S2 Secondary structure prediction for BnaCAT protein

Gene ID	α-helix	β-fold	Extended chain	Random curl
BnaC7.CAT1	55.05%	3.54%	12.63%	28.79%
BnaA8.CAT1	54.44%	3.69%	12.56%	29.31%
BnaC3.CAT1	53.27%	4.19%	13.40%	29.15%
BnaA3.CAT1	53.11%	3.36%	13.95%	29.58%
BnaC9.CAT2	41.69%	3.92%	16.14%	38.24%
BnaCn.CAT2	42.34%	4.58%	16.11%	36.97%
BnaA9.CAT2a	38.62%	4.24%	17.27%	39.87%
BnaA9.CAT2b	44.08%	4.74%	17.69%	33.49%
BnaA4.CAT3	39.78%	5.07%	18.86%	36.29%
BnaCn.CAT3	41.89%	5.83%	18.11%	34.17%
BnaA5.CAT4	43.88%	4.57%	15.66%	35.89%
BnaC5.CAT4	42.32%	3.59%	16.67%	37.42%
BnaC1.CAT4	47.76%	4.48%	14.59%	33.17%
BnaAn.CAT4	42.95%	3.98%	16.42%	36.65%
BnaAn.CAT5	53.86%	4.74%	13.86%	27.54%
BnaA4.CAT5	53.86%	4.74%	13.86%	27.54%
BnaA3.CAT6	52.65%	3.41%	15.34%	28.60%
BnaC7.CAT6	52.16%	3.63%	15.03%	29.19%
BnaC3.CAT6	52.65%	3.22%	15.15%	28.98%
BnaA9.CAT8	54.27%	4.10%	13.65%	27.99%
BnaC8.CAT8	54.53%	2.74%	13.33%	29.40%
BnaAn.CAT9	49.47%	5.44%	13.68%	31.40%
average	48.39%	4.17%	15.18%	32.26%
